# Supplementary material for: Promotion Effect of Coexposure to a High-Fat Diet and Nano-Diethylnitrosamine on the Progression of Fatty Liver Malignant Transformation into Liver Cancer
Source: Int J Mol Sci. 2023 Sep 15;24(18):14162. doi: 10.3390/ijms241814162 (PMC10531889; doi:10.3390/ijms241814162)
Supplement: Supplementary file 1 [file ijms-24-14162-s001.zip › Supplementary Material Table S1.pdf]

Supplementary Material Table S1.

HFD vs control		nanoDEN+HFD vs control		nanoDEN vs control	
**Rank**	**Description**	**Rank**	**Description**	**Rank**	**Description**
1	Pathways of neurodegeneration - multiple diseases	1	Pathways of neurodegeneration - multiple diseases	**1**	**FoxO signaling pathway**
2	Alzheimer disease	2	Chemical carcinogenesis - reactive oxygen species	2	PI3K-Akt signaling pathway
3	Amyotrophic lateral sclerosis	3	Non-alcoholic fatty liver disease	3	MicroRNAs in cancer
4	Chemical carcinogenesis - reactive oxygen species	4	PI3K-Akt signaling pathway	4	Pathways of neurodegeneration - multiple diseases
5	Thermogenesis	5	Human papillomavirus infection	5	**AMPK signaling pathway**
6	PI3K-Akt signaling pathway	6	Huntington disease	6	**p53 signaling pathway**
7	Human papillomavirus infection	7	Thermogenesis	7	Human papillomavirus infection
8	Non-alcoholic fatty liver disease	8	Alzheimer disease	8	Amyotrophic lateral sclerosis
9	Herpes simplex virus 1 infection	9	Parkinson disease	9	MAPK signaling pathway
10	Prion disease	10	Prion disease	10	Lipid and atherosclerosis
11	Huntington disease	11	Salmonella infection	11	Chemical carcinogenesis - reactive oxygen species
12	Diabetic cardiomyopathy	12	MAPK signaling pathway	12	Alcoholic liver disease
13	Salmonella infection	13	Human immunodeficiency virus 1 infection	13	Alzheimer disease
14	MAPK signaling pathway	14	Endocytosis	14	Kaposi sarcoma-associated herpesvirus infection
15	Endocytosis	15	Viral carcinogenesis	15	Non-alcoholic fatty liver disease
**16**	**p53 signaling pathway**	16	Coronavirus disease - COVID-19	16	Chemical carcinogenesis - receptor activation
17	Parkinson disease	17	Amyotrophic lateral sclerosis	17	Human cytomegalovirus infection
18	Chemical carcinogenesis - receptor activation	18	Human T-cell leukemia virus 1 infection	18	Thyroid hormone signaling pathway
19	Coronavirus disease - COVID-19	**19**	**p53 signaling pathway**	19	Prion disease
20	NOD-like receptor signaling pathway	**20**	**AMPK signaling pathway**	20	Hepatitis B
21	Oxidative phosphorylation	21	Diabetic cardiomyopathy	21	Hepatitis C
22	Biosynthesis of cofactors	22	Chemical carcinogenesis - receptor activation	22	Hepatocellular carcinoma
23	Human immunodeficiency virus 1 infection	23	Epstein-Barr virus infection	23	Proteoglycans in cancer
24	Proteoglycans in cancer	24	Human cytomegalovirus infection	24	Regulation of actin cytoskeleton
25	Epstein-Barr virus infection	25	Tight junction	25	Epstein-Barr virus infection
26	Viral carcinogenesis	26	Protein processing in endoplasmic reticulum	26	Parkinson disease
27	Human T-cell leukemia virus 1 infection	27	Alcoholic liver disease	27	Huntington disease
28	Focal adhesion	28	Biosynthesis of cofactors	28	Apoptosis
29	Rap1 signaling pathway	29	Hepatocellular carcinoma	29	Apelin signaling pathway
30	Protein processing in endoplasmic reticulum	30	Proteoglycans in cancer	30	Measles
31	Regulation of actin cytoskeleton	31	Lipid and atherosclerosis	31	Prostate cancer
32	Ubiquitin mediated proteolysis	32	Regulation of actin cytoskeleton	32	cGMP-PKG signaling pathway
33	Cellular senescence	33	Herpes simplex virus 1 infection	33	Viral carcinogenesis
34	Neutrophil extracellular trap formation	34	Insulin signaling pathway	34	Ras signaling pathway
35	Tight junction	35	Phagosome	35	Endocytosis
36	Autophagy - animal	36	Spliceosome	36	Platinum drug resistance
37	mTOR signaling pathway	37	Tuberculosis	37	Glucagon signaling pathway
**38**	**FoxO signaling pathway**	38	Lysosome	38	Insulin resistance
39	Hepatocellular carcinoma	39	Hepatitis C	39	Toxoplasmosis
40	Transcriptional misregulation in cancer	40	Axon guidance	40	Estrogen signaling pathway
41	Lysosome	41	Neutrophil extracellular trap formation	41	Insulin signaling pathway
42	Retrograde endocannabinoid signaling	42	Rap1 signaling pathway	42	Tight junction
43	Ras signaling pathway	43	Oxidative phosphorylation	43	Protein processing in endoplasmic reticulum
**44**	**AMPK signaling pathway**	44	Apoptosis	44	Tuberculosis
45	Hepatitis C	45	Necroptosis	45	NOD-like receptor signaling pathway
46	Wnt signaling pathway	46	Ras signaling pathway	46	Rap1 signaling pathway
47	Influenza A	47	MicroRNAs in cancer	47	Thermogenesis
48	Carbon metabolism	48	Measles	48	Human immunodeficiency virus 1 infection
49	Fluid shear stress and atherosclerosis	**49**	**FoxO signaling pathway**	49	Coronavirus disease - COVID-19
50	Pathways of neurodegeneration - multiple diseases	50	Pathways of neurodegeneration - multiple diseases	50	Human T-cell leukemia virus 1 infection

Note: Top 50 signaling pathways according to enrichment score. Compared with the control group, respectively. The red font represents oncogenic signaling pathways, while the blue font represents cancer suppressor related pathways (Figure 6).
