# Supplementary material for: Promotion Effect of Coexposure to a High-Fat Diet and Nano-Diethylnitrosamine on the Progression of Fatty Liver Malignant Transformation into Liver Cancer
Source: Int J Mol Sci. 2023 Sep 15;24(18):14162. doi: 10.3390/ijms241814162 (PMC10531889; doi:10.3390/ijms241814162)
Supplement: Supplementary file 1 [file ijms-24-14162-s001.zip › Supplementary Materials Figures S1--S4.pptx]

## Slide 1
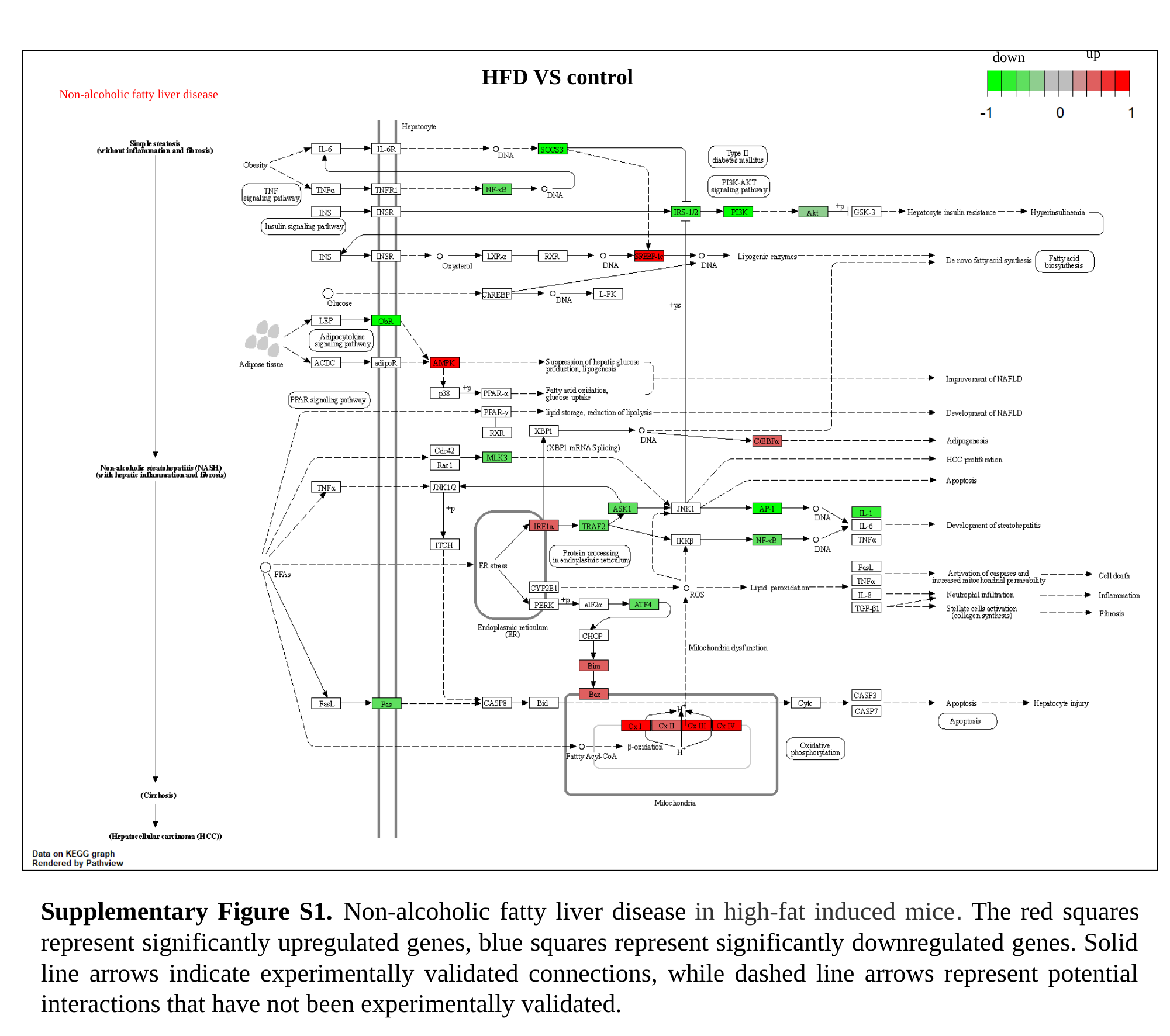

HFD VS control
Non-alcoholic fatty liver disease
up
down
Supplementary Figure S1. Non-alcoholic fatty liver disease in high-fat induced mice. The red squares represent significantly upregulated genes, blue squares represent significantly downregulated genes. Solid line arrows indicate experimentally validated connections, while dashed line arrows represent potential interactions that have not been experimentally validated.

## Slide 2
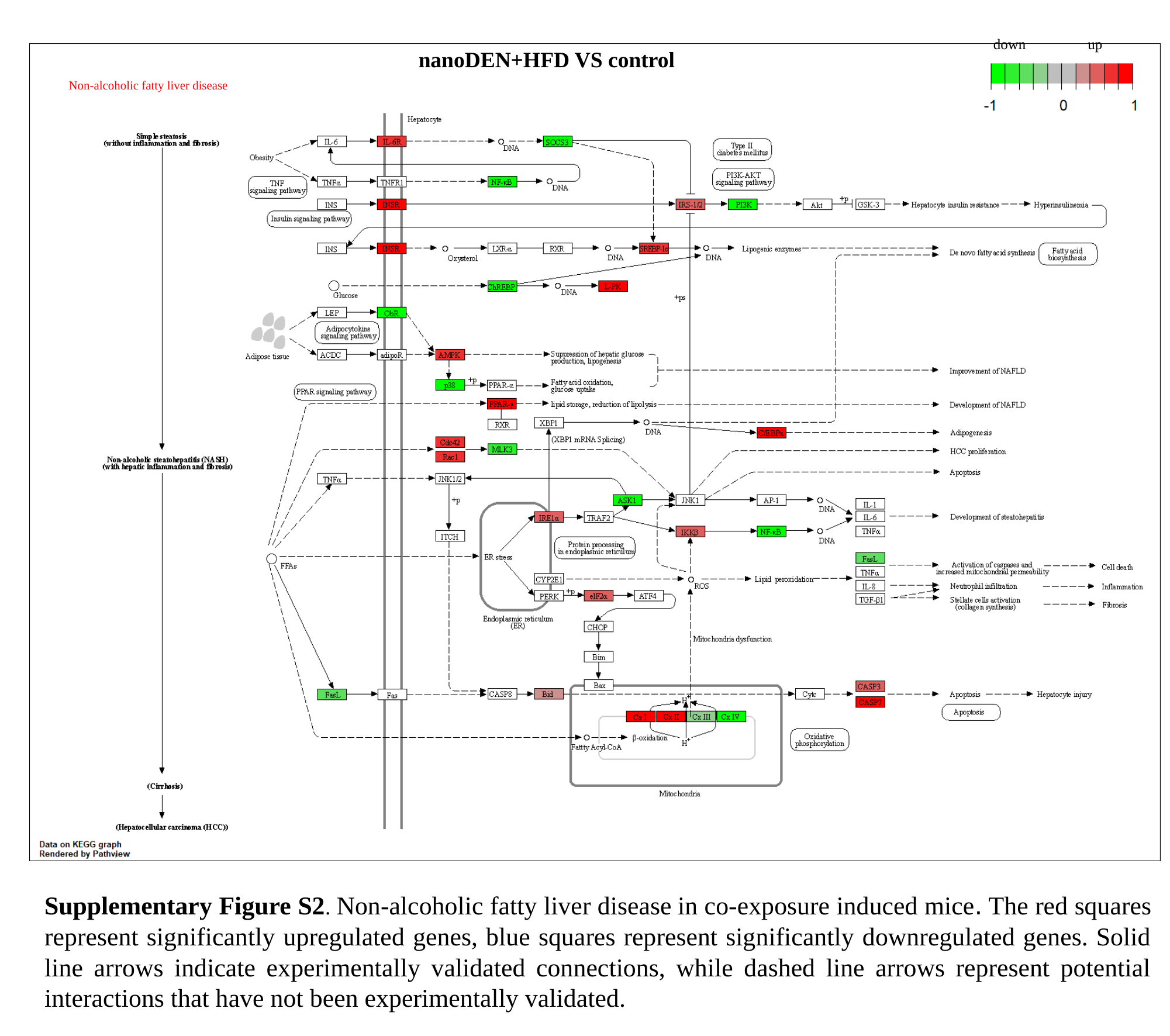

down
up
 nanoDEN+HFD VS control
Non-alcoholic fatty liver disease
Supplementary Figure S2. Non-alcoholic fatty liver disease in co-exposure induced mice. The red squares represent significantly upregulated genes, blue squares represent significantly downregulated genes. Solid line arrows indicate experimentally validated connections, while dashed line arrows represent potential interactions that have not been experimentally validated.

## Slide 3
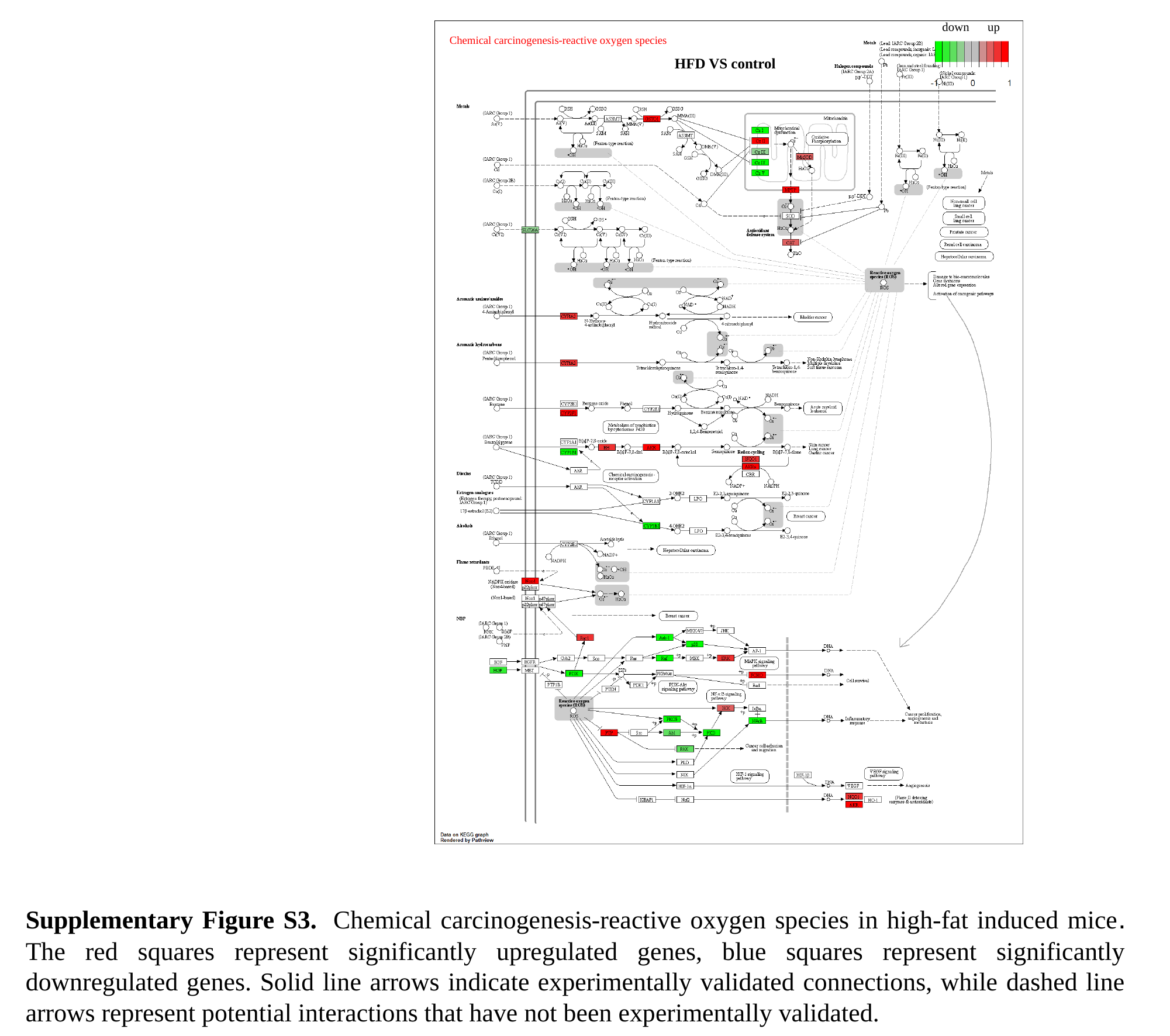

down
up
Chemical carcinogenesis-reactive oxygen species
HFD VS control
Supplementary Figure S3. Chemical carcinogenesis-reactive oxygen species in high-fat induced mice. The red squares represent significantly upregulated genes, blue squares represent significantly downregulated genes. Solid line arrows indicate experimentally validated connections, while dashed line arrows represent potential interactions that have not been experimentally validated.

## Slide 4
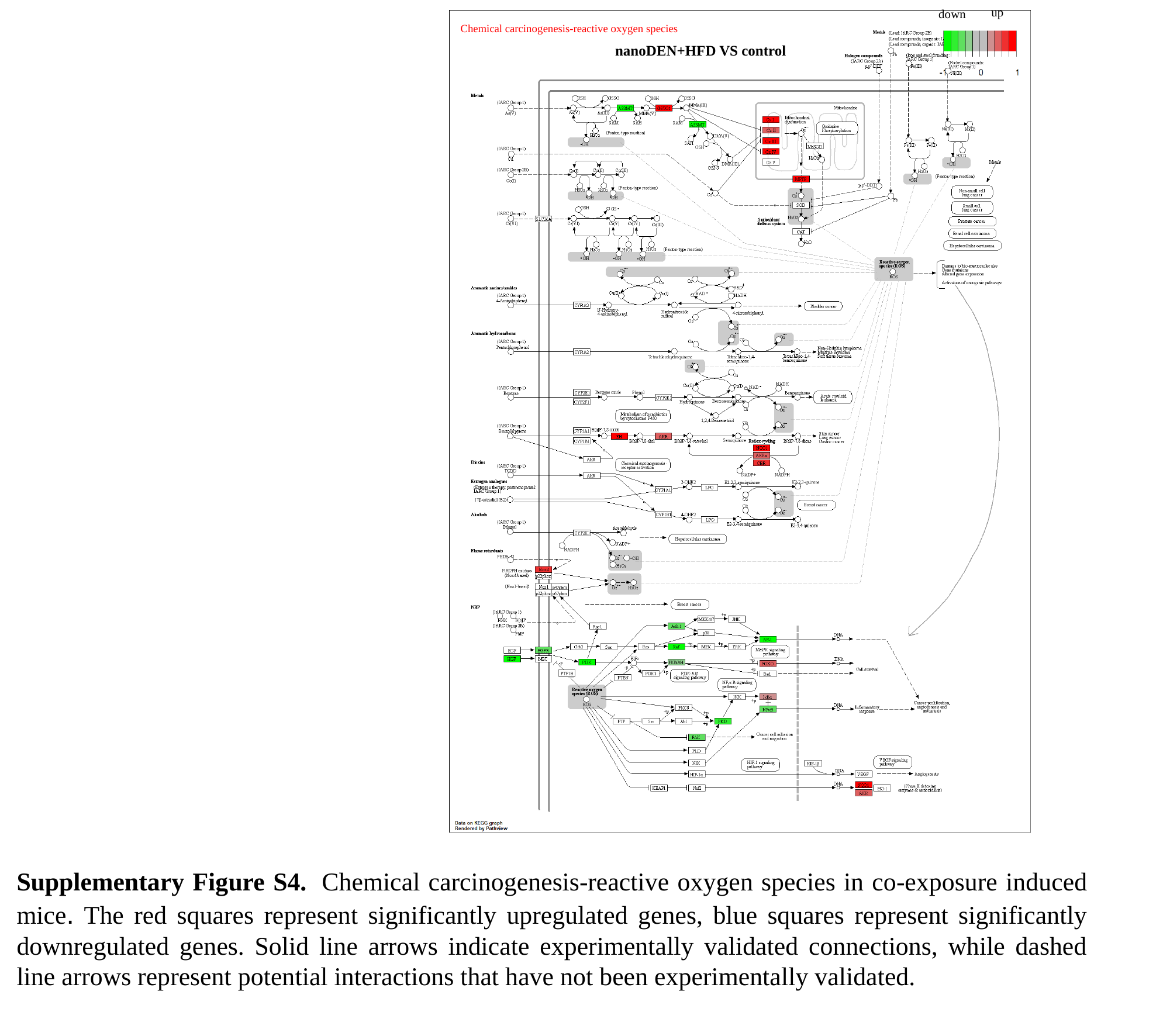

up
down
Chemical carcinogenesis-reactive oxygen species
nanoDEN+HFD VS control
Supplementary Figure S4. Chemical carcinogenesis-reactive oxygen species in co-exposure induced mice. The red squares represent significantly upregulated genes, blue squares represent significantly downregulated genes. Solid line arrows indicate experimentally validated connections, while dashed line arrows represent potential interactions that have not been experimentally validated.
